# The use of herbal medicines by people with cancer: a qualitative study

**DOI:** 10.1186/1472-6882-9-14

**Published:** 2009-05-14

**Authors:** Christine Gratus, Sue Wilson, Sheila M Greenfield, Sarah L Damery, Sally A Warmington, Robert Grieve, Neil M Steven, Philip Routledge

**Affiliations:** 1 Primary Care Clinical Sciences, The University of Birmingham, Birmingham, B15 2TT, UK; 2 Arden Cancer Centre, University Hospitals Coventry and Warwickshire, Clifford Bridge Road, Coventry, CV2 2DX, UK; 3 Cancer Research UK Clinical Trials Unit, University of Birmingham, Birmingham, B15 2TT, UK; 4 Department of Pharmacology, Therapeutics and Toxicology, Cardiff University, Heath Park, Cardiff, CF14 4XN, UK

## Abstract

**Background:**

Between 7% and 48% of cancer patients report taking herbal medicines after diagnosis. Because of the possibility of unwanted side effects or interactions with conventional treatments, people with cancer are generally advised to tell the professionals treating them if they are taking any form of medication, including herbal medicines and supplements. Studies suggest that only about half do so and that the professionals themselves have at best very limited knowledge and feel unable to give informed advice. This study is intended to inform the future development of information resources for cancer patients, survivors and healthcare professionals including tools for use before or during consultation to make it easier for patients to mention, and for healthcare professionals to ask about, use of herbal medications.

**Methods/design:**

This is a three-phase study. In phase 1, a systematic review of the literature on self-medication with herbal medicines among UK populations living with cancer will establish the current evidence base on use of herbal medicine, sources of information, characteristics and motivations. This will allow us to better understand what aspects need further investigation and inform the topic guide for a qualitative study (phase 2). Six focus groups of six to eight cancer patients who have used at least one herbal preparation since diagnosis will explore behaviour, beliefs, knowledge, information sources and needs in an informal conversational setting.

Informed by the findings of the systematic review and qualitative study, in phase 3 we will construct and pilot a questionnaire for a future large-scale survey to quantify and prioritise people's beliefs, needs and information preferences.

**Discussion:**

Despite known interactions with conventional cancer treatments and contraindications for some herbal remedies with specific cancers, reliable information resources for patients are very limited. Identifying cancer patients' information needs and preferences is the first step in creating a suitable resource for both the public and the professionals advising them.

## Background

Self-medication with herbal remedies and 'natural' medications taken by mouth is widespread and growing in the UK [1]. Almost half of women with breast cancer report taking herbal remedies, vitamins, or other supplements during treatment [2]. Among cancer patients in general, 7% report taking herbal medicines [3]. Self-medication may be used to help counteract the effects of cancer treatment; to alleviate symptoms of cancer; to boost the immune system; to deal with another condition; or in the hope of tackling the cancer itself [2,4,5]. It may also provide a sense of control or of being actively involved in treatment [3,5]. However, some herbal medicines may cause problems unrelated to cancer; black cohosh, used by women taking hormonal medications after breast cancer, is associated in rare cases with serious liver problems [6]. This herb is also contra-indicated for women with oestrogen-receptor positive breast cancer because of uncertainty about possible oestrogenic activity, as are Dong Quai and ginseng which have been shown to stimulate cell growth in a human breast cancer cell line. [7]. Herbal remedies can also interfere with conventional treatments. St John's Wort can speed up the time that the body takes to get rid of the anti-cancer treatment, Imatinib by 44% [8]. People nevertheless tend to believe that 'natural' remedies are harmless [3,5]. A study of patients on warfarin indicated that only 28% of those also taking herbal medicines believed that these could interact with conventional drugs [9].

Because of the possibility of unwanted side effects or interactions, people with cancer are generally advised to tell the professionals treating them if they are taking any form of medication, including herbal medicines and supplements [10]. Studies suggest that only about half of them do so [3,10] and that healthcare professionals themselves may be unable to give informed advice. A survey of anaesthetists in the UK revealed that although 65% felt that herbal medicines might sometimes have potentially harmful effects in people undergoing surgery, 82% felt that their own knowledge of herbal remedies was inadequate [11]. There is evidence that the most common sources of advice on herbal medications are friends and relatives or support and self-help groups [12].

There is no authoritative, readily accessible and independent source of information in the UK about herbal remedies and other supplements where people can find out about possible adverse (or beneficial) effects or interactions with their prescribed medicines. The NHS Direct website [13] offers superficial information on a limited number of conditions such as eczema and migraine, but fails to mention herbal remedies either in relation to cancer or to possible adverse effects or interactions with conventional drugs. Cancerbackup [14] offers a general warning that *'Some herbal medicines may contain substances that could be harmful to some people with cancer, such as arsenic, steroids or oestrogen*' with some advice in relation to specific herbs and Traditional Chinese Medicine (TCM) under Frequently Asked Questions. Cancer Research UK's CancerHelp website advises *'Do let your doctor know about any herbal remedies you are thinking of taking, or are already using' *[15]. The Medicines and Healthcare Products Regulatory Agency (MHRA) [16] is developing a series of patient information pages based on approved patient information leaflets from registered herbal medicines, but at present there are only ten such leaflets, and the site is not likely to be a natural port of call for cancer patients seeking information, nor is it specifically designed for them. By contrast, many herbal medicine retailers have a web presence and are actively advocating the use of a range of herbal treatments for cancer. Work among breast and prostate cancer patients does show that very few patients trust the Internet as a source of information or support, whether biomedical or non-biomedical [17]. Patients are concerned about not being able to judge the qualifications of the people making claims and thus being unable to judge the truth of the claims of those trying to promote Complementary and Alternative Medicine (CAM) or biomedicine [18]. Accessible, interpretable and reliable information materials regarding herbal medicines need to be developed.

This study will explore what people living with cancer believe about, and hope to achieve by, self-medication with herbal remedies; how far they are aware of the potential for harm in herbal self-medication; where they obtain information on herbal remedies they use to support their cancer treatment; if they seek help from healthcare professionals about herbal medicines, and, if so, why and from whom; what response and advice they receive; what kinds of information they would like, and how this could best be provided. By understanding the beliefs and motivations involved, this study will assist the development of educational materials for both patients and professionals including a herbal medications information sheet for use in routine clinical visits to encourage both disclosure by patients and questioning by healthcare professionals. It will also provide the necessary understanding and background for a subsequent quantitative study that will identify the most commonly used herbal preparations and help to prioritise information needs and appropriate ways of meeting them.

## Methods/Design

**Figure 1:**
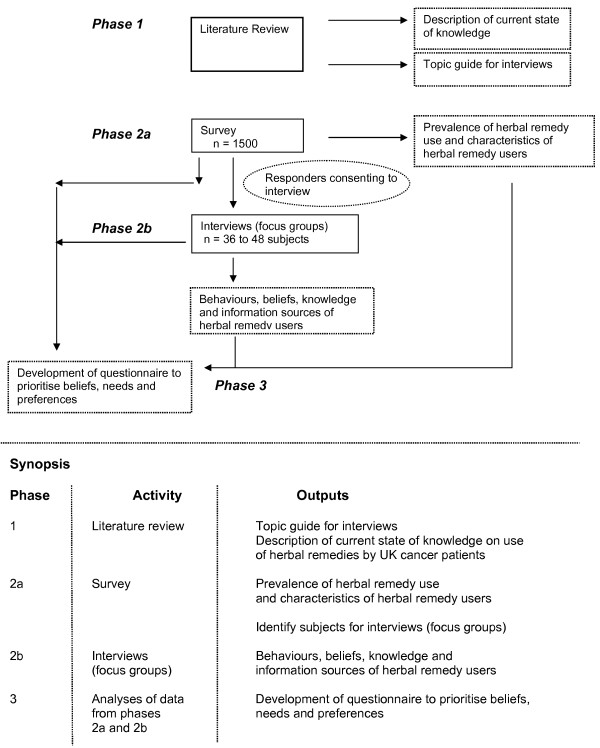
**Study design**.

This is a three-phase study incorporating a systematic review of the literature (phase 1), a qualitative study (phase 2) and the development and piloting of a questionnaire (phase 3) which will form the basis of a future survey. See figure 1. Study design.

### Phase 1: Literature review

A narrative systematic review of the literature on self-medication with herbal medicines and supplements among people living with cancer in the UK will be undertaken to establish what is known about how many use herbal remedies, what they take, their characteristics, motivations and information needs and sources. This will allow us to better understand where further investigation is required.

A scoping search of the literature suggests that research in this area is limited. The review will be confined to studies based on UK populations because practices and beliefs about herbal remedies may be very different in other countries. In Germany, for instance, 50% of phytomedicinal products are sold on medical prescription, the cost being refunded by health insurance [19]. In the UK, herbal medicine is seen as outside mainstream medical practice [1]. Both China and India have highly developed systems of traditional medicine based largely on herbs, and the beliefs and principles underlying these, (as well as the types of herbal remedies used), are very different from the biomedical model prevalent in the UK [20].

European Directive 2004/24/EC [21] defines a herbal medicinal product as 'any medicinal product, exclusively containing as active ingredients one or more herbal substances or one or more herbal preparations, or one or more such herbal substances in combination with one or more such herbal preparations'. A herbal substance is defined as 'whole, fragmented or cut plants, plant parts, algae, fungi, lichen in an unprocessed, usually dried, form, but sometimes fresh and herbal preparations'. Herbal preparations are defined as 'preparations obtained by subjecting herbal substances to treatments such as extraction, distillation, expression, fractionation, purification, concentration or fermentation'.

For the purposes of the literature review, herbal remedies will be defined as medicines conforming to the European definition above and taken for the treatment or prevention of signs and symptoms of disease or ill-health, or the maintenance of well-being. Herbal remedies made from more than one plant will be included, as will ginger, garlic and similar plant-derived substances if they are being used for medicinal purposes and are being taken in the form of supplements rather than as flavouring agents. Homeopathic preparations, minerals, and vitamins will be excluded. Electronic bibliographic databases (Medline, PsycLIT CINAHL, EmBase, AMED, SIGLE, NLH and other specialist databases) and web-based search strategies will be used.

A minimum of two independent reviewers will screen titles and abstracts for relevance. Disagreements will be resolved by discussion or after retrieval and examination of full papers. Data will be extracted by two reviewers using predefined forms, and papers will be quality assessed using a checklist. We anticipate that the variety of study designs and outcomes will prohibit meta-analysis and in this case synthesis will comprise a narrative review of the participants, interventions and outcomes.

### Phase 2: Qualitative study

#### Eligibility criteria

1. People who are at least six months and no more than five years after diagnosis of invasive cancer, who are being followed up at University Hospital, Birmingham NHS Foundation Trust or University Hospitals Coventry and Warwickshire Foundation Trust, and treated with curative intent. Recruitment will be via a postal screening questionnaire (see below). Patients known to be terminally ill, or whom the responsible consultant believes may be distressed by receipt of the questionnaire for any reason, will be excluded to minimise the possibility of distress.

2. People who are at least six months and no more than five years after diagnosis of invasive cancer, being followed up at either of the two participating hospitals and currently undergoing palliative treatment. Patient recruitment will take place during follow-up visits. This population will ensure that herbal remedy use to ameliorate the side-effects of palliative treatments such as chemotherapy and radiotherapy are included.

#### Recruitment

A screening questionnaire, letter of invitation and a patient information sheet will be sent to 1,500 eligible patients, 750 from each hospital site. The screening questionnaire will also be distributed, at follow-up appointments, to 200 patients who are known to be receiving palliative care. The questionnaire will establish patients' use of herbal medicines, socio-demographics, the nature and stage of their cancer, and their willingness to participate in a focus group. It will allow us to broadly describe the characteristics of users and provide information to enable us to undertake stratified recruitment to the focus groups.

In the unlikely event that insufficient participants for focus groups are identified by the screening questionnaire, a poster describing the study (and distribution of recruitment materials by research nurses at outpatient follow-up clinics in the two participating centres (Birmingham and Coventry) will be used as a 'back-up' recruitment strategy.

If any non-English-speaking patients express an interest in participating, the appropriate NHS Trust will be approached to provide an interpreter, and those patients will be interviewed individually.

#### Invitations to focus groups

Patients returning a completed screening questionnaire, having used at least one herbal medicine or supplement since diagnosis, and expressing an interest in further participation will be eligible to be invited to participate in a focus group. Invitees will be those who have either almost finished treatment, or have completed it recently enough to be able to remember what medications (herbal and conventional) they took.

Recruitment of 200 patients undergoing palliative care will enable differences between this population and the curatively treated group that form the basis of the survey to be identified. Should significant demographic or cancer-specific differences be found between those receiving curative and palliative treatment, we will attempt to hold a focus group of individuals from this sub-group.

Eligible patients will be contacted and arrangements made for them to attend a focus group to discuss their knowledge, behaviour, attitudes and beliefs in relation to self-medication with herbal remedies. Our previous experience indicates that participants are often more forthcoming in the presence of others of similar age, and we will ensure as far as possible that men are adequately represented, although they tend to use herbal remedies less often than women (77% women: 23% men)[22]. Where possible, we will have separate focus groups for people who are disease-free and who have progressive/longer term stable disease, as mixing patients at different stages of the disease may inhibit discussion. We will select participants accordingly, and replace refusals with others with similar characteristics. If some subsets of interest (e.g. working age men) remain under-represented, we will undertake mini-groups of 3–4 people, paired interviews or provide the option of individual interviews for those who want to contribute but cannot attend at the times allocated.

#### Feasibility

The prevalence of herbal medicine use among cancer patients has been estimated to be between 7% [3] and 13% [23]. Assuming a 60% response rate, a survey of 1,500 individuals would generate 900 responses; if 50% agree to participate in a focus group (n = 450) the number of respondents taking herbal medicines and thus eligible for interview will be between 31 (7%) and 58 (13% estimate). We would anticipate recruiting at least another ten eligible respondents from each site via additional recruitment activities (poster and distribution of materials by nurses). Up to 60 people will be invited to attend for interview. This will allow for six focus groups of six to eight people, plus any individual interviews as required to replace drop-outs and include people who may not be able to attend a group.

#### Power calculations

Assuming that the prevalence of herbal medicine use among cancer patients is between 7% [3] and 13% [23] and a 60% response rate, a survey of 1,500 individuals would generate 900 responses. This sample would be of sufficient size to determine the overall prevalence of herbal medicine use amongst respondents with a precision of 2% (95% confidence).

## Methods

### Content and conduct of focus groups

Focus groups are an effective way of exploring the views of people with a common background [24] as they allow interaction and discussion between participants. The focus group topic guide will be informed by existing literature and will cover a range of themes:

• use of herbal medication before and after cancer diagnosis

• reasons for use of herbal remedies (e.g. 'boost immune system', counteract effects of treatment)

• beliefs about the nature, properties and effects of herbal remedies

• whether use of herbal remedies was/is disclosed to healthcare professionals

• reasons for non-disclosure

• sources of advice (formal, informal, professional, commercial)

• role of healthcare professionals in providing advice on herbal medicine

• what advice was received/acted upon

• awareness/knowledge of and/or beliefs about possible side-effects and interactions with conventional medicines or effects on cancer itself

• changes in behaviour and beliefs as the patient journey progresses.

The topic guide will be structured around these themes, but with flexibility to explore any relevant issues that emerge from individual focus groups.

Experienced qualitative researchers will conduct the focus groups, with one researcher facilitating the group, and another observing and taking detailed field notes. The sessions will last one and a half to two hours, and will be recorded and fully transcribed. The groups will be held in locations convenient for participants.

### Analysis

Each transcript will be independently analysed by two experienced qualitative researchers, using the principles of thematic content analysis [25]. This involves reading the transcripts and identifying emerging themes and categories, with attention to the interaction between participants. Additionally, the co-applicants from different disciplines and two lay collaborators will read and analyse some of the (anonymised) transcripts in order to establish consensus about the key themes arising from the focus groups. These agreed themes will then be compared in order to detect similarities within, and differences between, groups. The emerging themes will be used to inform the design of the quantitative questionnaire for phase 3.

### Phase 3: Development of quantitative questionnaire

A questionnaire to quantify and prioritise people's beliefs, needs and preferences, suitable for both paper and electronic completion, will be developed based on the findings of the systematic review, the recruitment questionnaire and the qualitative study. It will be piloted on paper in ten face- to-face interviews conducted by the lay researchers, who will sit with respondents as they complete the questionnaire and identify omissions, problems of understanding and design issues. Members of the National Cancer Research Institute's Consumer Liaison Group (NCRI CLG) will also be asked to pilot the questionnaire online and to comment on its construction and content. The revised questionnaire will be re-tested with five face-to-face interviews.

### Ethical approval

This study has been approved by the Birmingham, East, North and Solihull Research Ethics Committee (REC), [Ref 08/H1206/153].

## Discussion

Most research into cancer patients' use of, and attitudes to, herbal medicines in the UK is carried out in the context of CAM generally. It is difficult to unravel the beliefs and information needs about complementary and alternative therapies with little potential for harm, such as reflexology or homeopathy, from those about herbal remedies, which have the potential both for harm and good. Some herbal medications can be dangerous, both in their own right and by interacting with cancer treatments. Despite this, there is no information readily available about the herbal remedies that people living with cancer use during and after their treatment, nor on the beliefs, motivations, knowledge and behaviour involved. Reliable information resources for patients, especially in the UK, are very limited. This study will assist the development of easily accessible educational materials for both patients and professionals, help to establish the kinds of information people would find useful, and indicate the most appropriate ways of ensuring that they can find it easily. It will inform the subsequent development of a quantitative survey that will help decision-makers prioritise both the information needed and the best ways to deliver it for relevance, ease of understanding and accessibility.

## Abbreviations

CINAHL: Cumulative Index to Nursing and Allied Health Literature; AMED: Allied and Alternative Medicine Database; SIGLE: System for Information on Grey Literature in Europe; NLH: National Library for Health Complementary and Alternative Medicine Specialist Database; EmBase: Excerpta Medica database; PsycLIT: Pyschological abstracts database.

## Competing interests

The authors declare that they have no competing interests.

## Authors' contributions

CG conceived the study. All the authors contributed to the development of the ideas and the design of the study. CG, SW, SG and SaW wrote the first draft of the protocol, which has been commented on by the other authors. All the authors read and approved the final manuscript.

## Pre-publication history

The pre-publication history for this paper can be accessed here:


